# Marginal measures and causal effects using the relative survival framework

**DOI:** 10.1093/ije/dyz268

**Published:** 2020-01-18

**Authors:** Elisavet Syriopoulou, Mark J Rutherford, Paul C Lambert

**Affiliations:** 1 Biostatistics Research Group, Department of Health Sciences, University of Leicester, Leicester, UK; 2 Department of Medical Epidemiology and Biostatistics, Karolinska Institutet, Stockholm, Sweden

**Keywords:** Relative survival, marginal measures, causal effects, avoidable deaths

## Abstract

**Background:**

In population-based cancer survival studies, the event of interest is usually death due to cancer. However, other competing events may be present. Relative survival is a commonly used measure in cancer studies that circumvents problems caused by the inaccuracy of the cause of death information. A summary of the prognosis of the cancer population and potential differences between subgroups can be obtained using marginal estimates of relative survival.

**Methods:**

We utilize regression standardization to obtain marginal estimates of interest in a relative survival framework. Such measures include the standardized relative survival, standardized all-cause survival and standardized crude probabilities of death. Contrasts of these can be formed to explore differences between exposure groups and under certain assumptions are interpreted as causal effects. The difference in standardized all-cause survival can also provide an estimate for the impact of eliminating cancer-related differences between exposure groups. The potential avoidable deaths after such hypothetical scenarios can also be estimated. To illustrate the methods we use the example of survival differences across socio-economic groups for colon cancer.

**Results:**

Using relative survival, a range of marginal measures and contrasts were estimated. For these measures we either focused on cancer-related differences only or chose to incorporate both cancer and other cause differences. The impact of eliminating differences between groups was also estimated. Another useful way for quantifying that impact is the avoidable deaths under hypothetical scenarios.

**Conclusions:**

Marginal estimates within the relative survival framework provide useful summary measures and can be applied to better understand differences across exposure groups.

**Key Messages**

- Marginal measures provide population estimates in a relative survival framework with a simple interpretation.
- Such measures are marginal relative survival, marginal all-cause survival and marginal crude probabilities of death.
- Under certain assumptions, the difference in marginal estimates between subgroups can be interpreted as the average causal effect.
- Using the relative survival framework enables us to focus on cancer-related differences instead of all-cause differences that are more difficult to explore.
- The avoidable deaths under hypothetical scenarios can also be estimated.

## Introduction

In population-based cancer survival studies the event of interest is usually death due to a specific cancer. However, death from other causes may prevent the event of interest occurring, i.e. there are so-called competing risks. To quantify cancer survival while accounting for differential other cause probability of death, net survival can be estimated. Net survival is the survival in a hypothetical world where the only possible cause of death is death due to cancer. It can be estimated using either the cause-specific or relative survival (excess mortality) approach.^1^^,^^2^ The cause-specific approach estimates cancer survival by censoring patients who died from other causes at the time of death. Information on cause of death is often inaccurate, particularly for the elderly. Relative survival is an alternative method to estimate net survival, but does not require information on cause of death through incorporating the expected mortality rates using population life tables.^3–7^ Information on appropriate expected mortality rates is essential for the interpretation of relative survival as net survival.

Within the relative survival framework, two probabilities of interest are the net probability of death, which is 1 minus relative survival, and the crude probability of death. Each approach yields a different estimand and the choice is based on the research question of interest.^8^ Net probabilities of death focus on a hypothetical world where the cancer of interest is the only possible cause of death. They provide a useful measure when comparing populations, such as countries or socio-economic groups, with differential other cause mortality rates as they focus on differences that are only due to the cancer of interest. Crude probabilities of death are more appropriate when making clinical decisions for a specific patient as they quantify survival in the presence of other possible causes of death.^9^ In the competing risks literature, the terminology is generally different where crude probabilities are often referred to as cause-specific cumulative incidence functions.^10^

To improve understanding of the mechanisms that drive associations, causal inference methods can be applied.^11^^,^^12^ The mathematical framework used for formulating statistical models and assumptions for causal inference is that of potential outcomes: the outcomes that would be observed if the patient received a specific level of the exposure.^13^ Causal effects are defined as contrasts of marginal effects of the potential outcomes and enable quantification of differences in prognosis of subgroups.^14^ In order to make causal statements certain assumptions need to hold and these are explored later in this paper in the context of the relative survival setting—with one main issue being the level of stratification in the lifetables that is adequate to achieve conditional exchangeability for other cause mortality. Marginal effects can be estimated using approaches such as inverse probability weights, but here our focus is on using regression standardization to obtain standardized survival and related functions.^15^^,^^16^ Their simple interpretation as a single measure for each time-point circumvents problems of communication of results from complex statistical models. Contrasts of standardized measures can also be utilized to investigate the impact of eliminating the observed differences between groups.

In this paper, we define various marginal measures using the relative survival framework and we define causal effects to compare population subgroups. To illustrate the measures we use the example of survival differences across socio-economic groups for colon cancer. Moreover, we estimate the potential avoidable deaths under a hypothetical scenario of eliminating survival differences between groups.

The remainder of the paper is organized as follows. First, we introduce the data to illustrate the methods and describe relative survival and excess mortality. Then, we define marginal measures of interest as well as contrasts between subgroups. Following, we describe contrasts within subsets of the population, including the avoidable deaths. Finally, we provide a summary of the methods.

## Introducing the illustrative example

Data, made available by Public Health England, includes patients diagnosed with colon cancer in 2008 in England, with follow-up to the end of 2013 and information on gender, age at diagnosis and deprivation status. There were five deprivation groups, derived from national quintiles of the income domain of the area of patients’ residence at diagnosis.^17^^,^^18^ For simplicity we only included the least and most deprived groups, resulting in 7346 patients in total, 55% of whom are in the least deprived group. More details on the study population are available in Table 1.

**Table 1.** Number of colon cancer patients (with proportions) for gender and age-groups by deprivation group

	Deprivation group	
	Least deprived	Most deprived
Gender		
Male	2136 (52.37%)	1772 (52.24%)
Female	1943 (47.63%)	1495 (45.76%)
Age group, years		
18–44	102 (2.50%)	116 (3.55%)
45–54	210 (5.15%)	209 (6.40%)
55–64	769 (18.85%)	521 (15.95%)
65–74	1149 (28.17%)	972 (29.75%)
75–84	1332 (32.66%)	1045 (31.99%)
85+	517 (12.67%)	404 (12.37%)

## Excess mortality and relative survival

The underlying all-cause mortality rate of an individual i, with covariate pattern $Z=z_{i}$, is given as the summation of the expected mortality rates if they did not have the cancer,$\;h^{*}\left(t|Z_{1}=z_{1i}\right),$ and their excess mortality due to the cancer, $\lambda \left(t|Z_{2}=z_{2i}\right)$:

$$h\left(t|Z=z_{i}\right)=h^{*}\left(t|Z_{1}=z_{1i}\right)+\lambda \left(t|Z_{2}=z_{2i}\right),$$

with Z denoting the set of all covariates.${\;Z}_{1}$ and $Z_{2}$ denote the covariates for expected and excess mortalities respectively. The expected mortality rate is obtained from available life tables on a comparable population in the general population matched by characteristics such as age, sex, calendar year and deprivation status.^2^ The survival analogue of excess mortality is relative survival. The relative survival of an individual, $i,\;R\left(t|Z_{2}=z_{2i}\right)$, is defined as their all-cause survival,$\;S\left(t|Z=z_{i}\right)$, divided by their expected survival, $S^{*}\left(t|Z_{1}=z_{1i}\right)$. The all-cause survival is thus given as

(1) $$S\left(t|Z=z_{i}\right)=S^{*}\left(t|Z_{1}=z_{1i}\right)R\left(t|Z_{2}=z_{2i}\right)$$

Relative survival accounts for different background mortality rates in patients with different characteristics, without having to rely on the cause of death information and under assumptions is interpreted as net survival.^7^^,^^19^ Net survival has the interpretation of the probability of survival in a hypothetical world where the only possible cause of death is the cancer of interest. In order for this interpretation to be valid certain assumptions need to hold. These are (i) appropriate expected mortality rate that represents mortality due to other causes for the cancer population and (ii) the potential times to death from cancer and other causes are conditionally independent. When important variables that affect both cancer and other causes of deaths are not included in the available life tables for the expected mortality rates, then comparability between populations is lost and relative survival cannot be interpreted as net survival.^19^

There are several models for relative survival and the following sections in theory can be applied to any of these.^20–23^ We choose flexible parametric survival models, which use splines to model the effect of time and are preferable in our setting as they incorporate time-dependent effects (non-proportional hazards) easily.^24^^,^^25^

For the illustrative example, we fitted a flexible parametric model with 5 degrees of freedom for the baseline excess hazard. The model included deprivation status, gender and age. Age was included as a continuous non-linear variable using restricted cubic splines with 3 degrees of freedom. We also included time-dependent effects for age and deprivation. We then derived various predictions based on the fitted model. More details on Stata code are available in Supplementary Appendix A, available as Supplementary data at *IJE* online.

## Marginal measures

Marginal measures provide population summaries with a simple interpretation.^16^ In the following subsections, we define the marginal relative survival function and describe how to obtain the marginal all-cause survival and marginal crude probabilities within the relative survival framework. Contrasts between subgroups are described in the section headed ‘Forming contrasts between population groups’.

### Marginal relative survival

Let $R\left(t\right|Z_{2})$ denote the conditional relative survival given covariates $Z_{2}$. The marginal relative survival is:

(2) $$\theta \left(t\right)=E[R\left(t\right|Z_{2})]$$

with the expectation over the marginal distribution of $Z_{2}$. After fitting a survival model, $\theta \left(t\right)$ can be estimated by obtaining predictions of relative survival for each individual in the study population and taking an average of these predictions,

$$\hat{\theta }\left(t\right)=\frac{1}{N}\sum_{i=1}^{N}\hat{R}\left(t|Z_{2}=z_{2i}\right)$$

where N is the number of patients in the population.

If interest is on the mortality scale, the standardized net probability of death can be obtained instead by $1-\hat{\theta }\left(t\right)$.

Standardizing to an external population might also be applied and is particularly common when comparing relative survival across different countries.^26^ For instance, the externally age-standardized relative survival is calculated as

$$\hat{\theta }\left(t\right)=\frac{1}{N}\sum_{i=1}^{N}w_{i}\hat{R}(t|Z_{2}{=z}_{2i})$$

where $w_{i}$ is the ratio of the proportion within an age group in the reference population to the corresponding group in the study population. Weights higher than 1 are applied to groups that are underrepresented in the study population compared with the standard population.^7^

### Marginal all-cause survival

To quantify survival in the presence of both cancer and other causes, the marginal all-cause survival function can be obtained. The estimand of interest in now defined as:

(3) $$\theta \left(t\right)=E[S\left(t\right|Z)]=E[S^{*}\left(t\right|Z_{1})R(t|Z_{2})]$$

and is estimated by the standardized all-cause survival

$$\hat{\theta }\left(t\right)=\frac{1}{N}\sum_{i=1}^{N}S^{*}\left(t|Z_{1}=z_{1i}\right)\hat{R}\left(t|{Z_{2}=z}_{2i}\right)$$

The standardized all-cause probability of death can also be estimated as $1-\hat{\theta }\left(t\right)$.

### Marginal crude probabilities of death

Let the crude probability of dying from the cancer of interest by time t in the presence of a competing risk of death due to other causes be $F_{c}\left(t\right|Z)$ and the probability of dying of causes other than the cancer of interest in the presence of cancer be $F_{o}\left(t\right|Z).$^27^ The marginal crude probability of dying from cancer is defined as

$$\theta_{c}\left(t\right)=E[F_{c}\left(t\right|Z)]=E\left[\int_{0}^{t}S^{*}\left(u|Z_{1}\right)R\left(u|Z_{2}\right)\lambda \left(u|Z_{2}\right)du\right]$$

and is estimated by the standardized crude-probability of death due to cancer

$${\hat{\theta }}_{c}\left(t\right)=\frac{1}{N}\sum_{i=1}^{N}\hat{F_{c}}\left(t\right|Z=z_{i})\;=\frac{1}{N}\sum_{i=1}^{N}\int_{0}^{t}S^{*}\left(u|Z_{1}=z_{1i}\right)\hat{R}\left(u|{Z_{2}=z}_{2i}\right)\hat{\lambda }(u|{Z_{2}=z}_{2i})\mathrm{du}\;$$

Similarly, the marginal crude probability of dying of causes other than the cancer of interest is estimated by

$${\hat{\theta }}_{o}\left(t\right)=\frac{1}{N}\sum_{i=1}^{N}\hat{F_{o}}\left(t\right|Z=z_{i})=\;\frac{1}{N}\sum_{i=1}^{N}\int_{0}^{t}S^{*}\left(u|Z_{1}=z_{1i}\right)\hat{R}\left(u|{Z_{2}=z}_{2i}\right)h^{*}(u|Z_{1}=z_{1i})\mathrm{du}\;$$

#### Example

**Figure 1.**
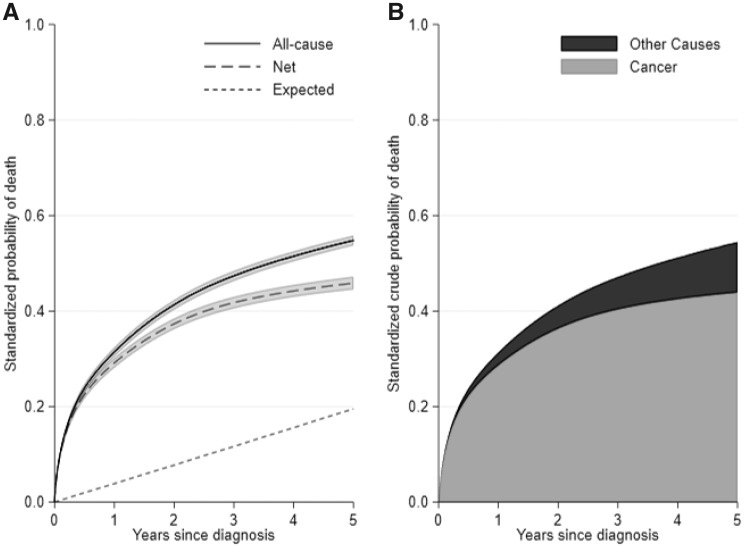
(**A**) Standardized all-cause and net probability of death, with 95% confidence intervals, as well as expected probability of death, and (**B**) stacked plot for the standardized crude probabilities for cancer and other causes, for the whole study population.

The marginal 5-year expected probability of death for a population without colon cancer is 20%. For the colon cancer population, the 5-year standardized net and all-cause probability of death was 46 and 55% respectively (Fig. 1). The net probability of death is lower as it is estimated in a hypothetical world where it is not possible to die from other causes. The all-cause probability of death can also be partitioned to that due to cancer and that due to other causes. The 5-year crude probability of death due to cancer in the presence of other risks was 44% and the crude probability of death due to other causes in the presence of cancer was 11% (Fig. 1).

## Forming contrasts between population groups

Let’s assume that we want to estimate the effect of exposure, X, on the time-to-event outcome, while allowing for confounding Z. For simplicity, *X* will be a binary variable with X=1 for the exposed patients and X=0 for the unexposed. Let $\theta \left(t|X=x,Z\right)\;$be the counterfactual survival function that we would have observed, had everybody in the population been exposed to level X=x.

To form contrasts between the exposure groups the difference can be estimated and it has the advantage of being collapsible.^28^^,^^29^ The difference between levels X=1 and X=0 is defined as $\theta \left(t|X=1,Z\right)-\theta \left(t|X=0,Z\right)$. The first term is the counterfactual survival function if everyone in our population had X=1 and the second term is the counterfactual survival function if everyone had X=0.

Contrasts between the counterfactual outcomes can be estimated using the observed outcomes, under some assumptions.^8^^,^^30^ These are (i) conditional exchangeability meaning that the outcome and the exposure are independent given covariates, (ii) consistency i.e. an individual’s potential outcome under a specific exposure corresponds to the actual outcome of this person under this exposure level and (iii) positivity so that the probability of being in every level of the exposure group is positive for all levels of *Z*. Notice that the assumptions are now extended to both outcomes (death due to cancer and death due to other causes). The conditional exchangeability assumption for the other cause mortality can only be achieved by adjusting the available life tables of the general population for sufficient variables (see Discussion for further details).

### Relative survival differences

The difference in marginal relative survival functions, comparing X=1 and X=0, is defined as

(4) $$E\left[R\left(t|X=1,Z_{2}\right)\right]-E\left[R\left(t|X=0,Z_{2}\right)\right]$$

and gives the difference in the hypothetical situation where the cancer of interest is the only possible cause of death. It is estimated by

$$\frac{1}{N}\sum_{i=1}^{N}\hat{R}\left(t|X=1,{Z_{2}=z}_{2i}\right)-\frac{1}{N}\sum_{i=1}^{N}\hat{R}\left(t|X=0,{Z_{2}=z}_{2i}\right).$$

Here everyone is first forced to be exposed (X=1) and then unexposed (X=0). A key point is that the average over confounders, $Z_{2}$, is the same when estimating both marginal effects.

### All-cause survival differences

The difference in marginal all-cause survival can also be defined by incorporating the expected survival in equation (4):

(5) $$E\left[S^{*}\left(t|X=1,Z_{1}\right)R\left(t|X=1,Z_{2}\right)\right]-E\left[S^{*}\left(t|X=0,Z_{1}\right)R\left(t|X=0,Z_{2}\right)\right]$$

and is estimated by

$$\frac{1}{N}\sum_{i=1}^{N}S^{*}\left(t|X=1,{Z_{1}=z}_{1i}\right)\hat{R}\left(t|X=1,{Z_{2}=z}_{2i}\right)-\frac{1}{N}\sum_{i=1}^{N}S^{*}\left(t|X=0,{Z_{1}=z}_{1i}\right)\hat{R}\left(t|X=0,{Z_{2}=z}_{2i}\right).$$

All-cause survival differences move away from the hypothetical world of relative survival and take into account both cancer-related and other-causes-related survival. Equation (5) has also the interpretation of the potential impact of removing all-cause differences between exposed and unexposed.

#### Example

**Figure 2.**
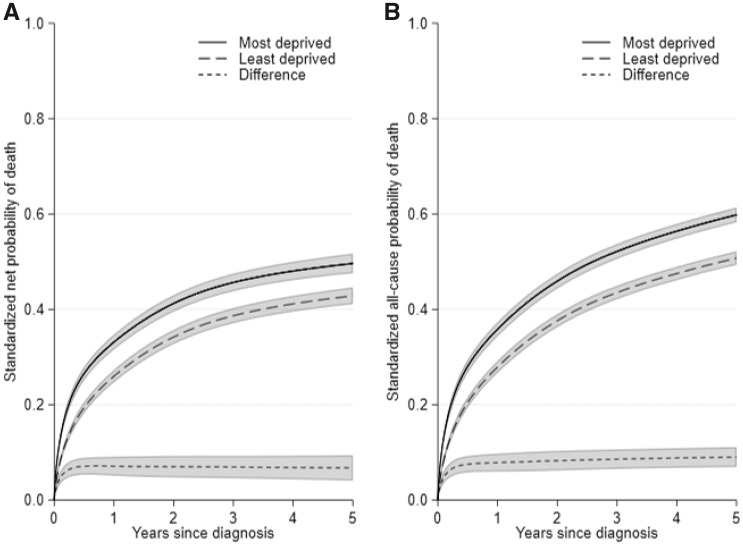
(**A**) Standardized net probability of death, and (**B**) standardized all-cause probability of death, for the least and most deprived patients with 95% confidence intervals.

Figure 2 shows the standardized net and all-cause probabilities of death by deprivation. These are standardized over the combined age and sex distribution. The 5-year standardized net probability of death of the least and most deprived group was 43 and 50% respectively. The 5-year standardized all-cause probability of death was 51 and 60% for the least and most deprived. However, such a comparison does not distinguish whether the difference is due to cancer mortality, other cause mortality or both.

## Forming contrasts within subsets of the population

It might also be of interest to estimate the measures and contrasts described earlier, within subsets of the whole population. For instance, the all-cause survival difference in the whole population, defined in equation (5), could also be defined among the exposed:

(6) $$E\left[S^{*}\left(t|X=1,Z_{1}^{X=1}\right)R\left(t|X=1,Z_{2}^{X=1}\right)\right]-E\left[S^{*}\left(t|X=0,Z_{1}^{X=1}\right)R\left(t|X=0,Z_{2}^{X=1}\right)\right]$$

with $Z_{1}^{X=1}$ and $Z_{2}^{X=1}$ denoting the covariates for the exposed, for the expected and relative survival respectively. It can be estimated by standardizing only to patients of the exposed group, $N^{X=1}$,

$$\frac{1}{N^{X=1}}\sum_{i=1}^{N^{X=1}}S^{*}\left(t|X=1,{Z_{1}^{X=1}=z}_{1i}\right)\hat{R}\left(t|X=1,{Z_{2}^{X=1}=z}_{2i}\right)-\frac{1}{N^{X=1}}\sum_{i=1}^{N^{X=1}}S^{*}\left(t|X=0,{Z_{1}^{X=1}=z}_{1i}\right)\hat{R}\left(t|X=0,{Z_{2}^{X=1}=z}_{2i}\right).$$

Forming contrasts within subsets of the population is useful when estimating the potential impact of removing differences for groups with worse survival, under hypothetical scenarios.

### Eliminating cancer-related differences

In practice, it might be difficult to remove all-cause survival differences as they are the result of complex mechanisms that involve both cancer-related and other cause mortality. A hypothetical scenario under which we eliminate cancer-related differences only may be easier to define. Contrasts of all-cause survival in which we only eliminate cancer-related survival differences between groups can be obtained using relative survival. For example, instead of equation (6), we could vary only relative survival between the two terms:

(7) $$E\left[S^{*}\left(t|X=1,Z_{1}^{X=1}\right)R\left(t|X=1,Z_{2}^{X=1}\right)\right]-E\left[S^{*}\left(t|X=1,Z_{1}^{X=1}\right)R\left(t|X=0,Z_{2}^{X=1}\right)\right]$$

which is estimated as

$$\frac{1}{N^{X=1}}\sum_{i=1}^{N^{X=1}}S^{*}\left(t|X=1,{Z_{1}^{X=1}=z}_{1i}\right)\hat{R}\left(t|X=1,{Z_{2}^{X=1}=z}_{2i}\right)-\frac{1}{N^{X=1}}\sum_{i=1}^{N^{X=1}}S^{*}\left(t|X=1,{Z_{1}^{X=1}=z}_{1i}\right)\hat{R}\left(t|X=0,{Z_{2}^{X=1}=z}_{2i}\right).$$

In equation (7), we assume that under the hypothetical scenario, the other cause mortality rate remains unchanged.

### Avoidable deaths

The impact of the hypothetical scenario described in the section ‘Eliminating cancer-related differences’ can also be estimated using avoidable deaths.^31^ Firstly, we need the predicted number of deaths for the exposed, which is given by multiplying the number of exposed patients diagnosed in a typical calendar year, $N^{*}$, with the probability of death:

$${D_{1}\left(t|X=1\right)=N}^{*}\left[1-E\left[S^{*}\left(t|X=1,Z_{1}^{X=1}\right)R\left(t|X=1,Z_{2}^{X=1}\right)\right]\right]$$

Secondly, we need the expected number of deaths under the hypothetical scenario that is derived by replacing the relative survival of the exposed with that of the unexposed:

$${D_{R_{0}}\left(t|X=1\right)=N}^{*}\left[1-E\left[S^{*}\left(t|X=1,Z_{1}^{X=1}\right)R\left(t|X=0,Z_{2}^{X=1}\right)\right]\right]$$

The avoidable deaths are then estimated by:

(8) $${{\mathrm{AD}}_{R_{0}}=D}_{1}\left(t|X=1\right)-D_{R_{0}}(t|X=1)$$

Each of the terms is estimated by

(9) $$N^{*}\left[1-\frac{1}{N^{X=1}}\sum_{i=1}^{N^{X=1}}S^{*}\left(t|X=1,{Z_{1}^{X=1}=z}_{1i}\right)\hat{R}\left(t|X=x,{Z_{2}^{X=1}=z}_{2i}\right)\right]$$

The number of exposed patients in a typical year, $N^{*},$ may be different from the patients we standardize over, $N^{X=1}$. For instance, $N^{*}$can be calculated by the number of exposed patients diagnosed in the most recent year or by the total number of exposed patients divided by the number of years.

Equation (8) yields the all-cause avoidable deaths among the exposed and can be partitioned to cancer or other causes deaths. This can be estimated by multiplying the marginal crude probabilities of death by the number of patients, $N^{*}$.

#### Example

**Figure 3.**
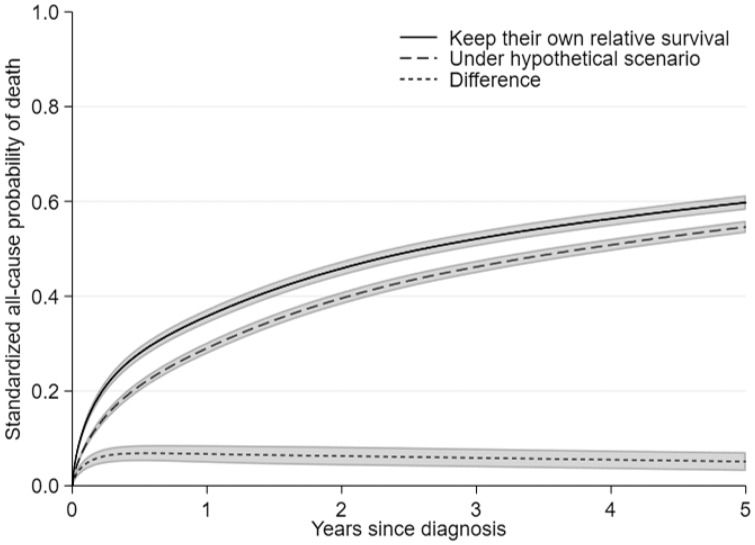
Standardized all-cause probabilities of death for the most deprived patients before and after the hypothetical scenario of removing differences in relative survival, with 95% confidence intervals.

We estimated the impact on the standardized all-cause probability of death of the most deprived group under a hypothetical scenario of removing differences in relative survival between deprivation groups. To do so, we applied the relative survival of the least deprived, i.e. the most advantaged group, to the most deprived, but kept their expected survival unchanged. In such a scenario the 5-year standardized all-cause probability of death of the most deprived would decrease from 60 to 55% (Fig. 3).

**Figure 4.**
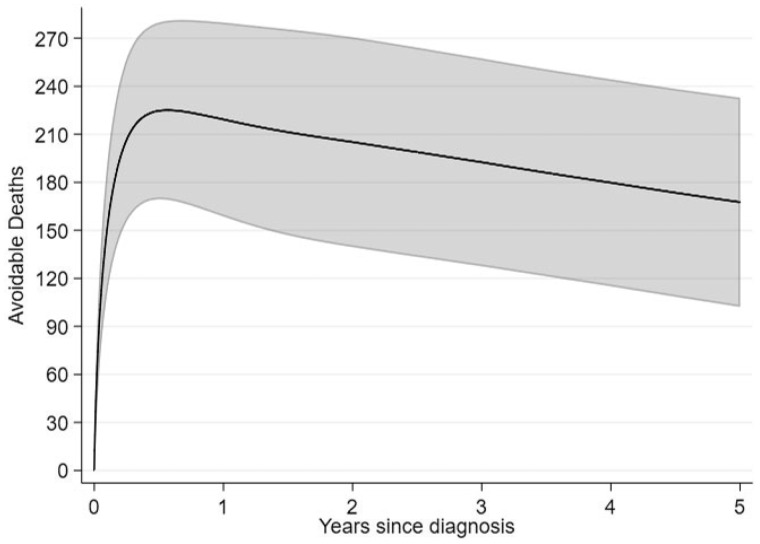
All-cause avoidable deaths under the hypothetical scenario of removing differences in relative survival between deprivation groups, with 95% confidence intervals.

**Figure 5.**
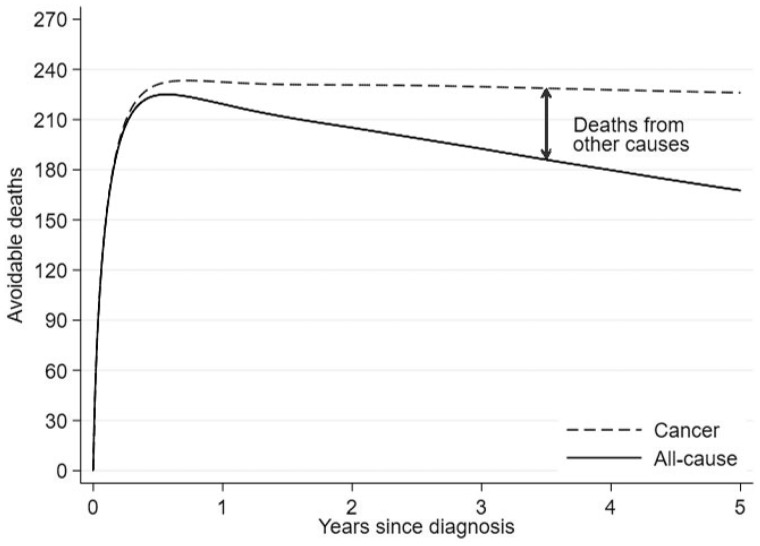
All-cause avoidable deaths partitioned to avoidable deaths due to cancer and increase in deaths due to other causes.

We also estimated the avoidable deaths for the most deprived patients under the same scenario. Five years after diagnosis 168 deaths could be avoided out of 3267 patients from the most deprived group diagnosed in 2008 (Fig. 4). In this example, $N^{*}$ and $N^{X=1}$ of equation (9) coincide (3267 patients) but this will not always be the case. Fig. 5 breaks down the all-cause avoidable deaths to cancer and other causes deaths. Even though the cancer avoidable deaths increase and finally stay constant with time, the all-cause avoidable deaths will decrease after the initial increase, as some patients that would die from the cancer will now die of other causes. That is why we observe an increase in other cause deaths.

## Discussion

We outlined marginal measures in a relative survival framework that can summarize the prognosis of a population. We also defined contrasts of these measures between subgroups of the population that under assumptions can be interpreted as causal effects. Most of these methods are used in practice, however in this paper we are formalizing them into a causal framework and providing software for their estimation. We also defined marginal crude probabilities as an additional useful measure. Marginal estimates were estimated using regression standardization. An advantage of these measures is that even after fitting a complex statistical model with interactions and time-dependent effects, a single number can be used to summarize the exposure effect at a given time point.^16^

In order to relate the counterfactual and the observed outcomes, some assumptions need to hold:^8^^,^^30^ conditional exchangeability, consistency and positivity. These have similar interpretation to that of an all-cause setting but here they are extended for both competing outcomes. Another assumption is that of well-defined interventions that would allow us to compute the causal effect in an ideal randomized experiment.^14^ As we evaluated the impact of an intervention that aims to eliminate deprivation differences one could argue that this is an ambiguous causal question. However, as others have argued, understanding the magnitude of disparities across deprivation groups in a formalized causal framework gives a firm basis to further unpick the reasons for the differences, even if the ideal randomized experiment would be difficult to precisely define.^12^^,^^32–35^ Our approaches can be extended to a mediation analysis setting to quantify measurable aspects that drive these inequalities, which will form part of future work.

In addition to standard causal inference assumptions, assumptions relevant to the relative survival need to hold. Information on expected mortality rates should be appropriate for the cancer population. Previous studies have assessed potential bias from including cancer patients in the general population. Bias was found to be negligible for individual cancers.^36–38^ Another assumption requires that the competing risks are conditionally independent.^19^ This means that there are no other factors to affect both competing events than the factors we have adjusted for. An example would be a strong effect of comorbidity that is likely to affect both cancer and other cause mortality rates.^39^ As the ability to adjust for confounders for other cause mortality depends on the available life tables, methods have been developed to do so when that information is not available.^40–42^ We appreciate that the estimates can only be interpreted as causal if this assumption is valid but, in principle, life tables can be constructed for any number of risk factors if there is available data to do so.

The interpretation of net survival in a hypothetical world where the only possible cause of death is the cancer of interest has received some criticism. Some proponents have argued that one should always ‘stick to this world’ for quantities of interest.^43^ Historically, relative survival has been used to account for differential mortality of competing events in population-based cancer survival.^2^^,^^44^ A hypothetical scenario of eliminating all-cause survival differences between exposures may not be straightforward in practice as many factors, which relate both to cancer and other causes, account for the differences. Relative survival allows to focus on cancer-related differences that may be easier to identify. Furthermore, we develop approaches for equalizing the excess mortality across population groups and then convert to ‘real’ world probabilities in the avoidable deaths measures we propose.

Avoidable deaths can be obtained to estimate the impact of eliminating survival differences between subgroups.^31^^,^^45^ The avoidable deaths depend on both the survival differences and the number of patients diagnosed with cancer and has the interpretation of postponable deaths as eventually all deaths will be observed. However, it helps to quantify the impact of removing survival inequalities for public health stakeholders.

We have demonstrated how to obtain marginal measures for the whole population or specific subsets as well as causal effects between exposure groups using the relative survival framework. Future work will focus on methods that will allow further investigation of observed differences. The relative survival framework could also be applied alongside mediation analysis to investigate the impact of potential mediators on the relationship between exposure and outcome.

## Supplementary Material

dyz268_Supplementary_Data
