# Erratum to: Marginal measures and causal effects using the relative survival framework

**DOI:** 10.1093/ije/dyaa032

**Published:** 2020-04-16

**Authors:** Elisavet Syriopoulou, Mark J Rutherford, Paul C Lambert

First published online: 18 January 2020, *Int J Epidemiol* 2020;49:619–28. doi: https://doi.org/10.1093/ije/dyz268

The originally published version of this article contained some errors in the setting of some equations (see below). These have now been corrected. OUP apologises for this error.

**Table:** 

Published version (wrong version)	How it should be (RIGHT version)
*Corrections in section: Marginal relative survival*	
*Page 3*	
$R\left(tZ_{2}\right)$	$R\left(t|Z_{2}\right)$
*Equation 2*	
$\theta \left(t\right)=E[R\left(tZ_{2}\right)]$	$\theta \left(t\right)=E\left[R\left(t|Z_{2}\right)\right]$
*Page 4*	
$\hat{\theta }\left(t\right)=\frac{1}{N}\sum_{i=1}^{N}\hat{R}(t||Z_{2}=z_{2i})$	$\hat{\theta }\left(t\right)=\frac{1}{N}\sum_{i=1}^{N}\hat{R}(t|Z_{2}=z_{2i})$
*Page 4*	
$\hat{\theta }\left(t\right)=\frac{1}{N}\sum_{i=1}^{N}w_{i}\hat{R}(t|,Z_{2}=z_{2i})$	$\hat{\theta }\left(t\right)=\frac{1}{N}\sum_{i=1}^{N}w_{i}\hat{R}(t|Z_{2}=z_{2i})$

*Corrections in section: Marginal all-cause survival*	
*Equation 3*	
$\theta \left(t\right)=E\left[S\left(tZ\right)\right]=E\left[S^{*}\left(tZ_{1}\right)R\left(t|Z_{2}\right)\right]\;\;\;$	$\theta \left(t\right)=E\left[S\left(t|Z\right)\right]=E\left[S^{*}\left(t|Z_{1}\right)R\left(t|Z_{2}\right)\right]\;\;\;$
*Page 4*	
$\hat{\theta }\left(t\right)=\frac{1}{N}\;\sum_{i=1}^{N}S^{*}\left(t|Z_{1}=z_{1i}\right)\hat{R}(t|{|Z}_{2}=z_{2i})$	$\hat{\theta }\left(t\right)=\frac{1}{N}\sum_{i=1}^{N}S^{*}\left(t|Z_{1}=z_{1i}\right)\hat{R}(t|Z_{2}=z_{2i})$

*Corrections in section: Marginal crude probabilities of death*	
*Page 4*	
$F_{c}\left(tZ\right)$	$F_{c}\left(t|Z\right)$
*Page 4*	
$F_{o}\left(tZ\right)$	$F_{o}\left(t|Z\right)$
*Page 4*	
$\theta_{c}\left(t\right)=E\left[F_{c}\left(tZ\right)\right]=E\left[\int_{0}^{t}S^{*}\left(u||Z_{1}\right)R\left(u||Z_{2}\right)\lambda \left(u||Z_{2}\right)du\right]$	$\theta_{c}\left(t\right)=E\left[F_{c}\left(t|Z\right)\right]=E\left[\int_{0}^{t}S^{*}\left(u|Z_{1}\right)R\left(u|Z_{2}\right)\lambda \left(u|Z_{2}\right)du\right]$
*Page 4*	
${\hat{\theta }}_{c}\left(t\right)=\frac{1}{N}\sum_{i=1}^{N}\hat{F_{c}}\left(tZ=z_{i}\right)\;=\frac{1}{N}\sum_{i=1}^{N}\int_{0}^{t}S^{*}(u||Z_{1}=z_{1i})\;\hat{R}\left(u||{Z_{2}=z}_{2i}\right)\hat{\lambda }(u|{Z_{2}=z}_{2i})du$	${\hat{\theta }}_{c}\left(t\right)=\frac{1}{N}\sum_{i=1}^{N}\hat{F_{c}}\left(t|Z=z_{i}\right)\;=\frac{1}{N}\sum_{i=1}^{N}\int_{0}^{t}S^{*}\left(u|Z_{1}=z_{1i}\right)\hat{R}\left(u|{Z_{2}=z}_{2i}\right)\hat{\lambda }(u|{Z_{2}=z}_{2i})du$

*Page 4*	
$\theta_{o}\left(t\right)=\frac{1}{N}\sum_{i=1}^{N}\hat{F_{o}}\left(tz_{i}\right)\;=\frac{1}{N}\sum_{i=1}^{N}\int_{0}^{t}S^{*}\left(u||Z_{1}=z_{1i}\right)\hat{R}\left(u|{Z_{2}=z}_{2i}\right)h^{*}(u|Z_{1}{=z}_{1i})du$	${\hat{\theta }}_{o}\left(t\right)=\frac{1}{N}\sum_{i=1}^{N}\hat{F_{o}}\left(t|Z=z_{i}\right)\;=\frac{1}{N}\sum_{i=1}^{N}\int_{0}^{t}S^{*}\left(u|Z_{1}=z_{1i}\right)\hat{R}\left(u|{Z_{2}=z}_{2i}\right)h^{*}(u|{Z_{1}=z}_{1i})du$
*Corrections in section: Relative survival differences*	
*Page 5*	
$\frac{1}{N}\sum_{i=1}^{N}\hat{R}\left(t||X=1,\;{Z_{2}=z}_{2i}\right)-\frac{1}{N}\sum_{i=1}^{N}\hat{R}\left(t||X=0,\;{Z_{2}=z}_{2i}\right)\;$	$\frac{1}{N}\sum_{i=1}^{N}\hat{R}\left(t|X=1,\;{Z_{2}=z}_{2i}\right)-\frac{1}{N}\sum_{i=1}^{N}\hat{R}\left(t|X=0,\;{Z_{2}=z}_{2i}\right)\;$
*Corrections in section: All-cause survival differences*	
*Equation 5*	
$E\left[S^{*}\left(t||X=1,\;Z_{1}\right)R\left(t|X=1,Z_{2}\right)\right]-E\left[S^{*}\left(t||X=0,\;Z_{1}\right)R\left(t|X=0,Z_{2}\right)\right]$	$E\left[S^{*}\left(t|X=1,\;Z_{1}\right)R\left(t|X=1,Z_{2}\right)\right]-E\left[S^{*}\left(t|X=0,\;Z_{1}\right)R\left(t|X=0,Z_{2}\right)\right]$
*Page 5*	
$\frac{1}{N}\sum_{i=1}^{N}S^{*}\left(t||X=1,\;Z_{1}=z_{1i}\right)\hat{R}\left(t||X=1,\;{Z_{2}=z}_{2i}\right)-\frac{1}{N}\sum_{i=1}^{N}S^{*}\left(t||X=0,\;Z_{1}=z_{1i}\right)\hat{R}\left(t||X=0,\;{Z_{2}=z}_{2i}\right)$	$\frac{1}{N}\sum_{i=1}^{N}S^{*}\left(t|X=1,\;Z_{1}=z_{1i}\right)\hat{R}\left(t|X=1,\;{Z_{2}=z}_{2i}\right)-\frac{1}{N}\sum_{i=1}^{N}S^{*}\left(t|X=0,\;Z_{1}=z_{1i}\right)\hat{R}\left(t|X=0,\;{Z_{2}=z}_{2i}\right)$

*Corrections in section: Forming contrasts within subsets of the population*	
*Equation 6*	
$E\left[S^{*}\left(t||X=1,Z_{1}^{X=1}\right)R\left(t|X=1,Z_{2}^{X=1}\right)\right]-E\left[S^{*}\left(t||X=0,Z_{1}^{X=1}\right)R\left(t|X=0,Z_{2}^{X=1}\right)\right]$	$E\left[S^{*}\left(t|X=1,Z_{1}^{X=1}\right)R\left(t|X=1,Z_{2}^{X=1}\right)\right]-E\left[S^{*}\left(t|X=0,Z_{1}^{X=1}\right)R\left(t|X=0,Z_{2}^{X=1}\right)\right]$
*Page 6*	
$\frac{1}{N^{X=1}}\sum_{i=1}^{N^{X=1}}S^{*}\left(t||X=1,\;{Z_{1}^{X=1}=z}_{1i}\right)\hat{R}\left(t||X=1,\;{Z_{2}^{X=1}=z}_{2i}\right)-\frac{1}{N^{X=1}}\sum_{i=1}^{N^{X=1}}S^{*}\left(t||X=0,\;{Z_{1}^{X=1}=z}_{1i}\right)\hat{R}\left(t||X=0,\;{Z_{2}^{X=1}=z}_{2i}\right)$	$\frac{1}{N^{X=1}}\sum_{i=1}^{N^{X=1}}S^{*}\left(t|X=1,\;{Z_{1}^{X=1}=z}_{1i}\right)\hat{R}\left(t|X=1,\;{Z_{2}^{X=1}=z}_{2i}\right)-\frac{1}{N^{X=1}}\sum_{i=1}^{N^{X=1}}S^{*}\left(t|X=0,\;{Z_{1}^{X=1}=z}_{1i}\right)\hat{R}\left(t|X=0,\;{Z_{2}^{X=1}=z}_{2i}\right)$

*Corrections in section: Eliminating cancer-related differences*	
*Equation 7*	
$E\left[S^{*}\left(t||X=1,Z_{1}^{X=1}\right)R\left(t|X=1,Z_{2}^{X=1}\right)\right]-E\left[S^{*}\left(t||X=1,Z_{1}^{X=1}\right)R\left(t|X=0,Z_{2}^{X=1}\right)\right]$	$E\left[S^{*}\left(t|X=1,Z_{1}^{X=1}\right)R\left(t|X=1,Z_{2}^{X=1}\right)\right]-E\left[S^{*}\left(t|X=1,Z_{1}^{X=1}\right)R\left(t|X=0,Z_{2}^{X=1}\right)\right]$
*Page 6*	
$\frac{1}{N^{X=1}}\sum_{i=1}^{N^{X=1}}S^{*}\left(t||X=1,\;{Z_{1}^{X=1}=z}_{1i}\right)\hat{R}\left(t||X=1,\;{Z_{2}^{X=1}=z}_{2i}\right)-\frac{1}{N^{X=1}}\sum_{i=1}^{N^{X=1}}S^{*}\left(t||X=1,\;{Z_{1}^{X=1}=z}_{1i}\right)\hat{R}\left(t||X=0,\;{Z_{2}^{X=1}=z}_{2i}\right)$	$\frac{1}{N^{X=1}}\sum_{i=1}^{N^{X=1}}S^{*}\left(t|X=1,\;{Z_{1}^{X=1}=z}_{1i}\right)\hat{R}\left(t|X=1,\;{Z_{2}^{X=1}=z}_{2i}\right)-\frac{1}{N^{X=1}}\sum_{i=1}^{N^{X=1}}S^{*}\left(t|X=1,\;{Z_{1}^{X=1}=z}_{1i}\right)\hat{R}\left(t|X=0,\;{Z_{2}^{X=1}=z}_{2i}\right)$

*Corrections in section: Avoidable deaths*	
*Page 7*	
${D_{1}\left(t||X=1\right)=N}^{*}\left[1-E\left[S^{*}\left(t||X=1,\;Z_{1}^{X=1}\right)R\left(t|X=1,Z_{2}^{X=1}\right)\right]\right]$	${D_{1}\left(t|X=1\right)=N}^{*}\left[1-E\left[S^{*}\left(t|X=1,\;Z_{1}^{X=1}\right)R\left(t|X=1,Z_{2}^{X=1}\right)\right]\right]$
*Page 7*	
${D_{R_{0}}\left(t||X=1\right)=N}^{*}\left[1-E\left[S^{*}\left(t||X=1,\;Z_{1}^{X=1}\right)R\left(t|X=0,Z_{2}^{X=1}\right)\right]\right]$	${D_{R_{0}}\left(t|X=1\right)=N}^{*}\left[1-E\left[S^{*}\left(t|X=1,\;Z_{1}^{X=1}\right)R\left(t|X=0,Z_{2}^{X=1}\right)\right]\right]$
*Equation 8*	
${{\mathrm{AD}}_{R_{0}}=D}_{1}\left(t||X=1\right)-D_{R_{0}}\left(t|X=1\right)$	${{\mathrm{AD}}_{R_{0}}=D}_{1}\left(t|X=1\right)-D_{R_{0}}\left(t|X=1\right)$
*Equation 9*	
$N^{*}\left[1-\frac{1}{N^{X=1}}\sum_{i=1}^{N^{X=1}}S^{*}\left(t||X=1,\;{Z_{1}^{X=1}=z}_{1i}\right)\hat{R}\left(t||X=x,\;{Z_{2}^{X=1}=z}_{2i}\right)\right]$	$N^{*}\left[1-\frac{1}{N^{X=1}}\sum_{i=1}^{N^{X=1}}S^{*}\left(t|X=1,\;{Z_{1}^{X=1}=z}_{1i}\right)\hat{R}\left(t|X=x,\;{Z_{2}^{X=1}=z}_{2i}\right)\right]$

