# Supplementary material for: Marginal measures and causal effects using the relative survival framework
Source: Int J Epidemiol. 2020 Jan 18;49(2):619–28. doi: 10.1093/ije/dyz268 (PMC7266533; doi:10.1093/ije/dyz268)
Supplement: dyz268_Supplementary_Data [file dyz268_supplementary_data.docx]

Appendix A

In this appendix, we provide the Stata code that was utilised to obtain the predictions of the paper. All predictions were obtained using command standsurv. You can download a pre-release version of standsurv within Stata using

net from https://www.pclambert.net/downloads/standsurv

In order to obtain predictions, we first need to declare the data as survival data and then we need to merge in the expected survival for the population life table (popmort.dta).

Age was modelled as a continuous non-linear variable using restricted cubic splines. This can be done with the following command:

rcsgen ageadj, df(3) gen(rcsa) orthog

The fitted model included age (splines), deprivation status and gender. We also allow for time-dependent effects for age and deprivation status:

stpm2 rcsa1 rcsa2 rcsa3 dep5 gender , df(5) scale(h) tvc(rcsa? dep5) ///

dftvc(3) bhaz(rate)

1. Marginal estimates of interest

To obtain standardized net probability of death (Figure 1.A):

standsurv, at1(.) timevar(timevar) atvars(stand_net) failure ci

The standardized all-cause probability of death & the standardised expected probability of death are derived in a similar way but now we need to also incorporate the expected survival using the expsurv option:

standsurv, at1(.) timevar(timevar) atvars(stand_obs) failure ci ///

expsurv(using(popmort.dta) ///

expsurvvars(expmort)

datediag(dx) ///

agediag(agediag) ///

pmrate(rate) ///

pmage(age) ///

pmyear(year) ///

pmother(dep sex) ///

at1(.))

For the standardized crude probabilities of deaths (Figure 1.B), we first need to define the function we are interested in:

mata function calc_allcause(at) return(at[1]+at[2])

and then use standsurv command to obtain predictions:

standsurv, at1(.) timevar(timevar) atvar(crprob) ///

crudeprob stub2(cancer other) ci ///

expsurv(using(popmort.dta) ///

datediag(dx) ///

agediag(agediag) ///

pmrate(rate) ///

pmage(age) ///

pmyear(year) ///

pmother(dep sex) ///

at1(.)) ///

userfunction(calc_allcause) ///

userfunctionvar(allcause)

1. Forming contrasts

The difference in standardised relative survival between the least and the most deprived patient groups (Figure 2.A) is obtained by:

standsurv, at1(dep5 0) at2(dep5 1) timevar(timevar) ///

contrast(difference) contrastvar(netdiff) ///

atvars(net1 net5) failure ci

The difference in standardised all-cause survival between the least and the most deprived patient groups (Figure 2.B) is obtained by the following command. Here we change both relative survival and expected survival.

standsurv, at1(dep5 0) at2(dep5 1) timevar(timevar) ///

contrast(difference)///

atvars(obs1 obs5) contrastvar(obsdiff) ///

failure ci ///

expsurv(using(popmort.dta) ///

datediag(dx) ///

agediag(agediag) ///

pmrate(rate) ///

pmage(age) ///

pmyear(year) ///

pmother(dep sex) ///

at1(dep 1 ) ///

at2(dep 5 ))

1. Forming contrasts within subsets of the population

The difference in standardised all-cause survival between the least and the most deprived patient groups if we change the relative survival but keep the expected survival unchanged is (Figure 3):

standsurv, at1(dep5 0, atif(dep5==1)) at2(dep5 1, atif(dep5==1)) ///

timevar(timevar) contrast(difference) ///

atvars(rs_changed rs_own) ///

contrastvar(obsdiff_changed) failure ci ///

expsurv(using(popmort.dta) ///

datediag(dx) ///

agediag(agediag) ///

pmrate(rate) ///

pmage(age) ///

pmyear(year) ///

pmother(dep sex) ///

at1(dep 5 ) ///

at2(dep 5 ))

1. Avoidable deaths

We want to estimate the avoidable deaths among the most deprived under a hypothetical scenario among the most deprived: what if the most deprived had the same relative survival as the least deprived group:

standsurv, at1(dep5 0 , atif(dep5==1)) at2(dep5 1 , atif(dep5==1)) ///

timevar(timevar) failure per(3267) ///

contrast(difference) contrastvar(ADa) ///

expsurv(using(popmort.dta) ///

datediag(dx) ///

agediag(agediag) ///

pmrate(rate) ///

pmage(age) ///

pmyear(year) ///

pmother(dep sex) ///

at1(dep 5) ///

at2(dep 5)) ci

To partition this further to cancer and other cause deaths:

standsurv , at1(dep5 0 , atif(dep5==1)) at2(dep5 1 , atif(dep5==1)) ///

timevar(timevar) crudeprob stub2(cancer other) per(3267) ///

contrast(difference) contrastvar(AD) nodes(125) ///

expsurv(using(popmort.dta) ///

datediag(dx) ///

agediag(agediag) ///

pmrate(rate) ///

pmage(age) ///

pmyear(year) ///

pmother(dep sex) ///

at1(dep 5) ///

at2(dep 5)) ci
